# Investigation of GPCR allosterism and dimerization in single living cells using fluorescent ligands

**DOI:** 10.1186/2193-1801-4-S1-L8

**Published:** 2015-06-12

**Authors:** Stehen Hill

**Affiliations:** Univerisity of Nottingham, UK

**Keywords:** Ligand binding, fluorescence, cooperativity

Previous work, using fluorescent adenosine receptor agonists and antagonists, has provided novel insights into the allosteric regulation of adenosine A3 (A3AR) and A1 (A1AR) receptors by allosteric ligands and receptor dimerization in single living cells [1, 2]. We have also used a fluorescent analogue of CGP12177 to investigate ligand binding to the human β1-adrenoceptor. This work has demonstrated that there is negative cooperativity between the two different ligand-binding conformations of the β1-adrenoceptor activated by catecholamines and CGP12177 respectively [3]. Finally, we have used fluorescence correlation spectroscopy (FCS) to investigate ligand binding to A1AR and A3AR in small 0.2 µm2 microdomains of single living cells [4]. FCS studies with a fluorescent A3-agonist have enabled high affinity labeling of the active conformation (R*) of the receptor [4]. We have also used a fluorescent adenosine A3-antagonist (CA200645) to study the binding characteristics of antagonist-occupied receptor conformations (R) in membrane microdomains of single cells [5]. Investigation of the dissociation kinetics of CA200645 provided further support for allosteric regulation of this receptor by homodimerization [5].
